# Clinical results of intradiscal hydrogel administration (GelStix) in lumbar degenerative disc disease

**DOI:** 10.3906/sag-1901-1

**Published:** 2019-12-16

**Authors:** Ayşegül CEYLAN, İbrahim AŞIK, G. Enver ÖZGENCİL, Burak ERKEN

**Affiliations:** 1 Department of Anesthesiology and Reanimation, Gülhane Training and Research Hospital, Ankara Turkey; 2 Department of Anesthesiology and Reanimation, Ankara University Medical Faculty Hospital, Ankara Turkey

**Keywords:** Degenerative disc disease, hydrogel, GelStix

## Abstract

**Background/aim:**

Degenerative disc disease (DDD) is one of the main causes of lower back pain. In this study, we evaluate the efficacy of percutaneous intradiscal GelStix administration in patients with discogenic pain due to lumbar DDD who were unresponsive to conservative methods.

**Materials and methods:**

A total of 29 patients were included in the study, which took place between 2013 and 2017. Sedation was performed in the prone position in the operating room, and a C-arm was located so as to provide a lateral view of the surgical field. A 22-G, 3.5-inch needle was inserted into the center of the disc under fluoroscopy guidance, and a percutaneous intradiscal GelStix implantation was performed. All patients were evaluated using the Oswestry Disability Index (ODI) and a visual analogue scale (VAS) before and after treatment, and using the Patient Satisfaction Scale at 12 months following treatment.

**Results:**

The mean VAS scores were 7.14 ± 0.64 at baseline and 2.48 ± 0.63 at 12 months (P < 0.001). The mean ODI scores were 28.14 ± 1.81 at baseline and 17.35 ± 0.67 at 12 months (P < 0.001). There was a statistically significant decrease in the VAS and ODI scores before and after treatment. A total of 86.2% of the patients rated the procedure as very good or good at 12 months.

**Conclusion:**

Our study results suggest that GelStix treatment is useful in pain relief in patients with DDD from the first month of treatment.

## 1. Introduction

Degenerative disc disease (DDD) is closely related with aging and lumbar back pain (LBP), which affects 70%–85% of the population during their lifetimes [1]. The prevalence of back pain increases with aging. Approximately 20% of young individuals have mild disc degeneration. The incidence of degeneration increases with aging, particularly in males; therefore, nearly 10% and 60% of discs in people aged 50 and 70 years, respectively, are severely degenerated [2]. 

The most important change caused by degeneration is the loss of proteoglycans (PGs). This may lead to a decrease in the osmotic pressure of the discal matrix and loss of hydration [3]. Furthermore, impaired nutrient transport in degenerated discs may lead to the formation of lactic acid and decreased pH levels [4]. There are many additional patient-specific factors that can contribute to the pathogenesis of the disease, and that have the potential to alter the natural course of disc degeneration. These include age, sex, genetics, smoking, cardiovascular disease, morbid obesity, physical inactivity, occupational factors (recurrent heavy lifting and vibration), constitutional weakness, low-grade discitis, spinal instability, and malignancy [5,6].

Hydrogels are water-soluble, and their hydrophilic features are suitable for minimally invasive spinal surgery, particularly surgery in which the hydrogel is transformed into a different form after implantation. In such cases, the minimized aqueous nature allows the implant to act as an interspinal expander [6].

There is a need for percutaneous interventional treatments between conservative and surgical methods that address the etiology of DDD but cause minimal damage to the annulus fibrosus (AF). It is difficult for the native nucleus pulposus (NP) to achieve self-renewal; however, hydrogels may serve as a substitute for the NP thanks to their hydrophilic and rheological properties and their similarities to native NP tissue. 

A hydrogel agent that ideally is injectable into the disc may improve the regeneration of NP and increase the mechanical function of the degenerative motional segment, particularly in patients with early or moderate degeneration who are unresponsive to conservative treatments [7,8]. In the present study, we evaluate the efficacy of percutaneous intradiscal GelStix administration in patients with discogenic pain due to lumbar DDD who were unresponsive to conservative methods.

## 2. Materials and methods 

### 2.1. Material selection

GelStix (Replication Medical, NCT02763956) is a modified filamentous version of polyacrylonitrile that enlarges in volume after implantation. As with previously used in situ hydration polymers, a number of complications have been reported, including disintegration of the gel after swelling [8]. Accordingly, NP-class polymers for NP implant hydration have been used with caution [9,10]. GelStix implants take the elongated hydrogel form of the registered polymer of RMI and are produced as matched bone that can be inserted under local anesthesia via 22-G needles. GelStix Nucleus Augmentation hydrates by absorbing the body’s own liquids, and its volume is expanded around tenfold and is reduced in less than 15 min.

### 2.2. Patient selection 

The inclusion criteria were as follows: being older than 18 years of age; being within American Society of Anesthesiologists Class I–II; having a visual analogue scale (VAS) score of ≥5/10 points; having discogenic pain due to 1 or 2 degenerative disc diseases; having a black disc, as confirmed by magnetic resonance imaging (MRI) compatible with clinical examinations; negative facet joint blocks and medial branch blocks; and having refractory symptoms despite physical therapy, muscle relaxant, and antiinflammatory treatment for at least 3 months. 

The exclusion criteria were as follows: patients with root compression or zygapophyseal arthrosis as documented by plain X-ray and lumbar MRI, and those with vertebral fractures, previous lumbar spine surgery, signs or symptoms of lumbar canal stenosis, psychological disorders, localized or systemic infections, tumors, coagulopathy, pregnancy, osteoarthritis-disc herniation-annular tear (Grade >4 Modified Dallas Grading), disc height of 5 mm or less than 50% of the original height, and a body mass index of ≥35 kg/m^2^.

Of the total patients involved in the study, 8 had middle lumbar axial pain (n = 8) and mild radiculopathy (n = 3). 

### 2.3. The procedure 

The study was approved by the Ethics Committee and was conducted in accordance with the principles of the Declaration of Helsinki. Written informed consent was obtained from each patient. The data of patients who were treated in our pain clinic with percutaneous intradiscal GelStix implantation for LBP between 2013 and 2017 were analyzed retrospectively, and data related to lumbar disc degeneration, medical history, physical examination, plain radiography, and MRI findings were recorded. 

The procedures were carried out under sterile conditions using fluoroscopy with a standard oblique intradiscal approach. Prior to the procedure, 1 g of intravenous cefazolin for prophylaxis and 2–5 mg of midazolam to reduce anxiety and discomfort were administered. The patients were thus calm but alert and conscious, and able to talk to the practitioner to report any unusual pain. During the procedure, blood pressure, heart rate, electrocardiography, oxygen saturation, and respiration rate were monitored for all patients.

The participants were placed in the prone position on the operating table, the operational area was cleaned and covered with sterile cloth, and an interventional point was identified under fluoroscopy and marked 8–10 cm laterally from the lumbar vertebrae on the side of intervention. A local anesthetic of 60 mg of prilocaine was injected into the subcutaneous tissues, and a 22-G, 3.5-inch needle was inserted into the center of the disc under fluoroscopy guidance. After entry was made, the position of the needle within the disc was checked via both anteroposterior and lateral views. Before installing the GelStix cartridge, liquids were removed using a stylus. The protective cap of the implant holder was removed and the holder was then pushed into the proximal end of the introducer needle and locked. It was confirmed that the tip of the needle was located at the center of the disc cavity by fluoroscopy, and the piston of the holder was pushed to allow the implant to completely pass the needle, with 3 implants placed at each disc level. Care was taken to avoid twisting the needle. While the needle tip was still at the center of the nucleus, the intradiscal area was washed out with 40 mg of gentamicin, an appropriate prophylactic local antibiotic. At the end of the procedure, the needle was pulled out and a sterile bandage was applied as a dressing. No sutures were used.

### 2.4. Postprocedural care 

All patients were allowed unlimited walking, standing, and sitting, and all were instructed to avoid heavy lifting, forward skin bending, or crushing. After 10 to 14 days, light work and home-based exercises with gentle flexions and extensions were allowed.

All patients were evaluated using the Oswestry Disability Index (ODI) and a VAS before treatment and at 1, 3, 6, and 12 months after treatment, and using the Patient Satisfaction Scale at 12 months following treatment.

### 2.5. Statistical analysis 

The data analysis was carried out using SPSS V 21.0 statistical software (IBM Corp., Armonk, NY, USA). Descriptive data were expressed as mean ± standard deviation (SD) for continuous variables and as number (n) and percentage (%) for nominal variables. The compatibility of the variables to normal distribution was checked with a Kolmogorov–Smirnov test. For abnormally distributed variables, intragroup distribution was compared using Friedman’s analysis of variance (ANOVA) test. If present, multiple intertime comparisons of the differences were evaluated using the Bonferroni adjusted Wilcoxon signed-ranks test. The power for nonparametric tests was unable to be calculated [11]. Clinical significance was assessed using Kendall’s W correlation coefficient for Friedman’s ANOVA and a correlation coefficient for the Wilcoxon signed-ranks test. Kendall’s W correlation coefficient was interpreted using Cohen’s guidelines of 0.1 (small effect), 0.3 (medium effect), and above 0.5 (strong effect). The effect size of the Wilcoxon signed-ranks test was interpreted according to Cohen’s criteria, and the values of 0.2, 0.5, and 0.8 were considered small, medium, and strong effect sizes, respectively. P < 0.05 was considered statistically significant. 

## 3. Results

A total of 29 patients were included in the study, and of these, 14 were female and 15 were male, with a mean age of 49.21 ± 6.82 years for women and 46.26 ± 7.18 years for men. All patients were followed for 12 months in the pain clinic. 

 GelStix implants were applied to 25 patients, to a single level in L4–L5 in 16 patients, to L5–S1 in 9 patients, and to 2 levels (L4–L5, L5–S1) in 4 patients. According to the Patient Satisfaction Scale, which was evaluated at 12 months following the procedure, 4 patients rated the procedure as very good, 21 as good, and 4 as moderate. Overall, 86.2% of the patients rated the procedure as very good or good (Table 1).

**Table 1:** Demographic data.

Sex	n	%
Male	15	51.7
Female	14	48.3
Level		
L4–L5	16	55.17
L5–S1	9	31.04
L4–S1	4	13.79
PSS		
Very good	4	13.8
Good	21	72.4
Moderate	4	13.8

PSS: Patient Satisfaction Score.

The mean VAS scores were 7.14 ± 0.64 at baseline, 3.69 ± 0.60 at 1 month, 2.93 ± 0.59 at 3 months, 2.62 ± 0.49 at 6 months, and 2.48 ± 0.63 at 12 months (Figure). Based on the results of a Friedman test, the differences between the VAS scores at baseline and at 1, 3, 6, and 12 months were found to be statistically significant (P = 0.001) (Table 2).

**Figure 1:**
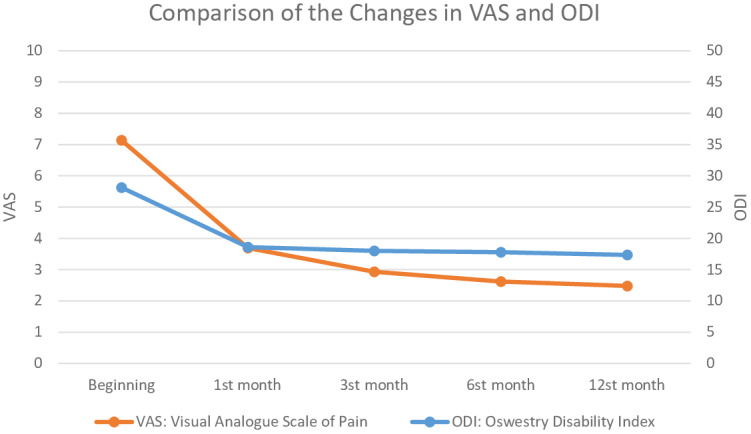
Comparison of the changes in VAS and ODI.

**Table 2:** The average of visual analogue score (VAS) results.

	VAS score(mean ± SD)	Test statistics*	Source of difference **
0	7.14 ± 0.64	χ2 = 93.078P < 0.001	0–1, 0–3, 0–6, 0–12 (P < 0.001)
1st month	3.69 ± 0.60		1–3, 1–6, 1–12 (P < 0.001)
3rd month	2.93 ± 0.59		3–6 (P = 0.029), 3–12 (P = 0.002)
6th month	2.62 ± 0.49		6–12 (P = 0.317)
12th month	2.48 ± 0.63		

*: Friedman variance analysis test statistical values. **: Bonferroni adjusted Wilcoxon signed-ranks test statistical values.

Kendall’s W value for the VAS was calculated as 0.802, which is higher than 0.5, indicating a strong effect size by Cohen’s criteria. This result was also clinically significant [11,12]. When comparing binary months, all of the differences between the VAS scores were statistically significant (P < 0.05), while only the difference between months 6 and 12 was not significant (P = 0.317). When the effect size of binary comparisons for the VAS scores was assessed according to Cohen’s criteria, the VAS scores for months 6–12 were below mild clinical significance, 3–6 about mild, and 3–12 between mild and moderate clinical significance, while the other time comparison results had more than moderate clinical significance [13] (Table 2; Figure). 

The differences in the ODI scores of the patients were found to be statistically significant (P = 0.001) using a Friedman test. The mean ODI scores were 28.14 ± 1.81 at baseline, 18.59 ± 1.84 at 1 month, 18.00 ± 1.10 at 3 months, 17.79 ± 1.01 at 6 months, and 17.35 ± 0.67 at 12 months. A decrease in the ODI scores in patients with GelStix treatment was noted (Figure). Kendall’s W value for the ODI scores was calculated at 0.635, which is higher than 0.5, indicating a strong effect size by Cohen’s criteria. This result was also clinically significant. When comparing binary months, the differences between the ODI scores were statistically significant (P < 0.05), except for the differences between the 1st and 3rd months (P = 0.110) and the 3rd and 6th months (P = 0.295). When the effect size of the binary comparisons of the ODI scores was assessed according to Cohen’s criteria, the ODI scores for months 3–6 had below mild clinical significance, 1–3 and 1–6 had about mild, and 3–12, 6–12, and 1–12 had between mild and moderate clinical significance, while the other time comparison results had more than moderate clinical significance (Table 3). 

**Table 3:** The average Oswestry Disability Index (ODI) scores.

	ODI score (mean ± SD)	Test statistics^*^	Source of difference ^**^
0	28.14 ± 1.81	χ^2^ = 93.078P < 0.001	0–1, 0–3, 0–6, 0–12 (P < 0.001)
1st month	18.59 ± 1.84		1–3 (P = 0.110), 1–6 (P = 0.038)
3rd month	18.00 ± 1.10		1–12 (P = 0.002), 3–6 (P = 0.295)
6th month	17.79 ± 1.01		3–12 (P = 0.003), 6–12 (P = 0.012)
12th month	17.35 ± 0.67		

^*^: Friedman variance analysis test statistical values. ^**^: Bonferroni adjusted Wilcoxon signed-ranks test statistical values.

## 4. Discussion

Disturbances of nutrient transport in degenerated discs cause lactic acid formation and decreased pH levels [4]. Accumulations of lactic acid modify cellular activity by downregulating PG synthesis, and it is the enzymes that degrade the extracellular matrix that cause Gelstix to stop the disk degeneration cycle. GelStix increases pH in the disc: low pH is associated with degeneration and inflammation, while increased pH causes the natural PGs to swell and increase hydration. Adding liquid and volume increases osmotic pressure, and low pH weakens the ability of PGs to bind to water; these factors lead to reduced hydration within the disc and further deterioration of the nutrient transport. 

The ideal hydrogel must meet the following requirements for NP renewal: i) it must be injectable, ii) it must prevent the cells or gel from escaping after implantation*, *iii) it must be strong and have adequate durability, iv) it must have sufficient distension pressure with various loads, v) it must support cell proliferation and matrix, and vi) it must prevent adverse effects [14]. A hydrogel should support cell growth and matrix, and should also have sufficient mechanical strength for NP renewal. 

In our study of patients with DDD, we observed a time-dependent decrease in the patients’ VAS and ODI scores with GelStix treatment, and the changes before and after the procedure indicated that the process contributed significantly to the reduction in VAS and ODI scores. At 12 months, the mean VAS scores had decreased from 7.14 at the beginning to 2.48 at the 12th month (P = 0.001), and the mean ODI scores had decreased from 28.14 to 17.35 (P = 0.001).

Singh et al. [15] administered GelStix implantations for 22 patients with DDD in 2012 and found the VAS scores to decrease from 8.5 to 3.0 (P < 0.0001) and the ODI scores to decrease from to 25.1 to 10.2 (P < 0.0001) after the first 4 weeks of treatment. In our study, the VAS and ODI scores of all patients with DDD at 12 months were similar to those in the aforementioned studies, and none of the patients in the study developed permanent neurological damage or complications requiring additional surgery. Response to treatment following GelStix implantation was noted early on in the treatment according to the selection of suitable patients and application by experienced operators. 

In patients with no history of severe radiculopathy, the rapid development of symptoms immediately after implantation suggests a possible misplacement at the annular ring [16]. In all patients, the cannula was carefully drained after it was clarified as being located most centrally in the nucleus pulposus via lateral and anteroposterior views from the C-arm.

GelStix can grow rapidly by absorbing water and can lead to an increase in radicular symptoms via root compression or can cause a protruding disc to exert pressure, resulting in direct radiculopathy. In 2 previous case reports, the medical history of the 2 patients involved revealed signs of disc compression and radiculopathy prior to the procedure, and their symptoms intensified after 4 and 6 months. This finding suggests that both patients had a broad-base protruding disc and a degenerated annulus fibrosis defect before the procedure [9,17]. 

The first-line treatment of DDD is conservative [18], and if conservative treatment fails, the current surgical treatment options are to surgically immobilize the degenerated disc segment, either with a fusion procedure or with a mechanical arthroplasty device [19,20]. 

It was not possible to compare the outcomes of our patients to results found in a systematic review of the conservative treatments for DDD [21], as the DDD patients were undergoing drug and physical therapy for general back pain. In the treatment of chronic LBP, multiple physical and rehabilitation interventions are effective in the short-term but ineffective in the long-term [22], and intensive and long-term physical therapy programs have been found to increase the severity of pain in certain cases [23]. Nonsteroidal antiinflammatory drugs (NSAIDs) and antidepressants with pain-relieving effects of opioids have been reported as having less pain-relieving effects in cases of chronic pain, and NSAIDs and opioids in particular have been reported as having serious side effects related to their long-term use [24].

### 4.1. Conclusions

The application of GelStix contributes to discal restoration in discogenic back pain and mild radicular pain, but it requires skilled practitioners as much as the selection of suitable patients due to the potentially serious complications. If the pain migrates towards the epidural space in the case of a newly developed injury or a preexisting weakness in the wall of the annular ring, the risk of complications and the need for surgery may increase due to severe radiculopathy. Based on the results of the present study, it can be suggested that GelStix intradiscal implant applications may be considered as a long-term pain-relieving, effective, and functionally beneficial treatment method for patients with DDD in experienced centers when based on appropriate patient selection.

### 4.2. Limitations 

In terms of evaluating the long-term results of Gelstix application after returning to routine daily life, the patients’ smoking habits and alcohol intake, weight loss or gain, occupational changes, and long-term quality of sleeping could have been detected individually and stated in the discussion. Thus, we could have had the chance to analyze the reasons for VAS and ODI changes during the control periods of 3 months. However, the data of the patients in our study were only collected at the beginning and specified follow-up times.
